# Patients predict their own outcome with CRT

**DOI:** 10.1007/s12471-015-0776-4

**Published:** 2015-11-19

**Authors:** M.B. Kronborg, J.C. Nielsen

**Affiliations:** Department of Cardiology, Aarhus University Hospital, Skejby, Palle Juul-Jensens Boulevard 99, 8200 Aarhus N, Denmark

Cardiac resynchronisation therapy (CRT) improves survival and reduces symptoms in patients with drug refractory heart failure and bundle branch block, and has been implemented into international guidelines over the last decade [1]. Despite the fact that several large randomised studies have demonstrated CRT to be very efficient, around 30–50 % of the patients receiving CRT – depending on what outcome is measured – do not benefit from this treatment strategy. These patients are often referred to as non-responders.

Potential candidates for CRT include some of the most severely symptomatic heart patients who have a poor prognosis and often multiple comorbidity including ischaemic heart disease, renal dysfunction, diabetes, and chronic obstructive pulmonary disorder. Although the success rate of establishing CRT has improved markedly, the risk of complications is still significantly higher than for other cardiac implantable electronic devices, which has to be taken into account in this fragile population [2]. As a consequence of that, a number of studies have focused on identifying baseline factors predicting which patients can expect to benefit from CRT to select the most appropriate candidates. These prognostic variables range from advanced cardiac imaging or invasive measurements, often expensive and time consuming acquiring highly specialised equipment and expertise, to more simple, inexpensive, and readily available information at every centre, such as baseline comorbidity and the standard 12-lead electrocardiogram. So far, none of the advanced measures have been generally accepted as superior to the more simple methods for detecting non-responders [3], and a further search for better prognostic markers is needed.

Versteeg et al. present the predictive value of pre-implant patient-reported health status on mortality using the Kansas City Cardiomyopathy Questionnaire (KCCQ) in a single-centre cohort of patients receiving CRT [4]. This well-conducted study demonstrated that a poor health status at baseline, defined as a KCCQ score of < 50 out of 100 points, was associated with a 2.5-fold increase in the combined endpoint of all-cause mortality or hospitalisation in a cardiology department during four years of follow-up. Furthermore, a poor health status was associated with a threefold increase in amount of days spent in hospital. The patient-reported health status was a stronger predictor than traditional baseline characteristics previously shown to have an impact on clinical outcome in CRT patients including renal dysfunction, New York Heart Association (NYHA) functional class, and male gender. With this comprehensive evaluation of the patients’ perception of their disease, the authors have brought focus on a very important aspect of patient care in CRT that is often overlooked. Commonly, physicians focus more on ‘objective’ technical measures or on how they interpret the patients’ health status.

This study indicates that the KCCQ may be an important tool to identify CRT candidates with a higher risk of adverse outcome. These patients may potentially benefit from more intensive follow-up including remote monitoring after implantation. The results of this study support inclusion of the KCCQ or other patient-reported outcomes in future observational and interventional studies in heart failure patients and patients with implanted cardiac devices [5]. Furthermore, since improvement in self-reported health status after implantation of a CRT device has been found to be associated with increased survival [6], the KCCQ might potentially also be a more relevant and more sensitive endpoint than echocardiographic parameters or NYHA classification when testing new strategies within the field of CRT. It is important not to focus only on length of survival but also on improvement of patient-reported outcomes and quality of life.

The sample size in the study by Versteeg et al. was moderate, and the confidence intervals wide. Therefore, before implementing these new tools in daily clinical practice the prognostic value of self-reported health scores should be confirmed in larger multicentre cohort studies with long follow-up to increase the power of the estimates and to allow more variables in the analyses, which would minimise the risk of residual confounding. Randomised controlled trials should be awaited before implementing new follow-up or treatment regimens for these patients with lower KCCQ scores. It would also be interesting to investigate how changes in the KCCQ score affect long-term outcome and whether any intervention that improves the patient-reported health status without directly altering the heart failure therapy – such as psychological training to cope better with a chronic disease – has any impact on long-term outcome in these patients.

## Funding

None.

## Conflict of interests

None declared.
